# Increased Adhesiveness of Blood Cells Induced by Mercury Chloride: Protective Effect of Hydroxytyrosol

**DOI:** 10.3390/antiox13121576

**Published:** 2024-12-20

**Authors:** Pasquale Perrone, Raquel Ortega-Luna, Caterina Manna, Ángeles Álvarez-Ribelles, Victor Collado-Diaz

**Affiliations:** 1 Department of Precision Medicine, School of Medicine, University of Campania “Luigi Vanvitelli”, 80138 Naples, Italy; pasquale.perrone@unicampania.it; 2 Departamento de Farmacología, Facultad de Medicina, Universidad de Valencia, 46010 Valencia, Spain; raquel.ortega-luna@uv.es (R.O.-L.); victor.collado@uv.es (V.C.-D.); 3 Centro de Investigación Biomédica en Red en Enfermedades Hepáticas y Digestivas (CIBERehd), 28029 Madrid, Spain

**Keywords:** hydroxytyrosol, mercury, toxicity, neutrophils, erythrocytes, adhesion

## Abstract

Mercury (Hg) is a highly toxic environmental contaminant that can harm human health, ultimately leading to endothelial dysfunction. Hg toxicity is partially mediated by the exposure of the cell membrane’s surface of erythrocytes (RBCs) to phosphatidylserine (PS). In the context of these challenges, hydroxytyrosol, a phenolic compound of olive oil, has the ability to mitigate the toxic effects of Hg. This study aims to analyze the effect of Hg on the adhesion of RBCs and polymorphonuclear cells (PMNs) to the vascular endothelium and the potential protective effect of hydroxytyrosol, as these interactions are crucial in the development of cardiovascular diseases (CVDs). RBCs, PMNs, and human vein endothelial cells (HUVECs) were treated with increasing concentrations of HgCl_2_ and, in some cases, with hydroxytyrosol, and their adhesion to HUVECs and the expression of adhesion molecules were subsequently analyzed. Our results demonstrate that HgCl_2_ significantly increases the adhesion of both RBCs (2.72 ± 0.48 S.E.M., *p*-value < 0.02) and PMNs (11.19 ± 1.96 S.E.M., *p*-value < 0.05) to HUVECs and that their adhesiveness is significantly reduced following treatment with hydroxytyrosol (RBCs, 1.2 ± 1.18 S.E.M., *p*-value < 0.02 and PMNs, 4.04 ± 1.35 S.E.M., *p*-value < 0.06). Interestingly, HgCl_2_ does not alter the expression of adhesion molecules on either HUVECs or RBCs, suggesting that reduced exposure to PS is a key factor in hydroxytyrosol protection against HgCl_2_-induced RBC adhesion to the endothelium. On the other hand, HgCl_2_ induces increased expression of several PMN adhesion molecules (CD11b 215.4 ± 30.83 S.E.M. *p*-value < 0.01), while hydroxytyrosol inhibits their expression (e.g., CD11b 149 ± 14.35 S.E.M., *p*-value < 0.03), which would seem to be the mechanism by which hydroxytyrosol restricts PMN–endothelium interactions. These results provide new insights into the molecular mechanisms through which hydroxytyrosol mitigates the harmful effects of Hg on cardiovascular health, highlighting its potential as a therapeutic agent that can reduce the cardiovascular risk related to heavy metal exposure.

## 1. Introduction

Mercury (Hg) is a highly toxic environmental pollutant whose detrimental effects on human health are well documented. As a pervasive heavy metal, Hg can enter the human body in various ways, for example, through inhalation, ingestion, and dermal absorption [1]. Blood Hg levels above 5–15 µg/L are considered to be toxic, especially for vulnerable populations such as pregnant women and children [2]. Once inside the body, Hg can produce a spectrum of toxic effects, including some affecting the circulatory and immune systems [3].

The molecular mechanisms underlying Hg toxicity are complex and not yet fully understood. One of the main reasons behind Hg toxicity is its strong affinity with the sulfhydryl groups of cellular thiols [4]. By reacting with crucial cysteine residues, Hg interacts with proteins, thus altering and inhibiting their enzymatic and structural functions, potentially leading to severe dysfunction of cellular activities. Additionally, Hg significantly reduces glutathione (GSH) levels, thereby compromising the antioxidant defense mechanisms of the cells [5].

Recent research has shown that blood cells, such as erythrocytes (RBCs) and leukocytes, are important ‘targets’ for the toxicity of Hg in humans. Hg preferentially accumulates in RBCs [6], inducing morphological changes but resulting in the exposure of phosphatidylserine (PS) on the outer membrane’s leaflet [5]. Under normal conditions, there is limited interaction between RBCs and endothelial cells, but RBC adhesion to the endothelium is facilitated when PS exposure increases [7]. In this context, recent studies have stressed the role of RBCs in cardiovascular disease (CVD) pathophysiology, highlighting their involvement in key processes such as inflammation, oxidative stress, thrombus formation, and the development of atherosclerotic plaques [8,9].

In addition to its effects on RBCs, Hg exposure also has a profound impact on the immune system. For example, Hg has been shown to increase the leukocyte count while decreasing platelet levels in rats, thereby altering the process of hemostasis [3]. The disruption of leukocyte–endothelial interactions is another important aspect of CVD pathophysiology and constitutes a critical step in the initiation and progression of atherosclerosis [10,11,12]. These interactions begin with leukocytes rolling along the endothelium until they can adhere firmly, a process mediated by different adhesion molecules present on leukocytes [such as PSGL-1, Mac-1 (CD11b/CD18), LFA-1 (CD11a/CD18), or L-selectin)] and on endothelial cells (E-selectin, P-selectin, intracellular adhesion molecule-1 (ICAM-1), or vascular cell adhesion molecule-1 (VCAM-1)] [13,14,15,16,17]. It has been shown that Hg enhances the adhesion of monocytes to endothelial cells [18]. However, there are no data to confirm an effect of Hg on the adhesion of polymorphonuclear cells (PMNs) to the endothelium or on the expression of adhesion molecules.

In response to growing concern over heavy metal toxicity, recent biomedical research has focused on natural substances that can mitigate these effects. Hydroxytyrosol, a polyphenol found in olive oil, has garnered significant attention due to its potent anti-inflammatory, antiproliferative, and antimicrobial properties, as well as its benefits for cardiovascular health [19]. Studies have demonstrated that hydroxytyrosol can reduce Hg toxicity in human RBCs by decreasing ROS generation and preventing GSH depletion [20]. Interestingly, hydroxytyrosol also limits PS exposure to RBCs [5,21].

The present study examines the effect of Hg on the adhesion of RBCs and PMNs to the endothelium with the aim of obtaining a clearer picture of the toxic influence of this heavy metal on the cardiovascular system. To determine the molecular mechanisms underlying these processes, the expression of various adhesion molecules is analyzed. Furthermore, to gain a more comprehensive understanding of the protective effect of hydroxytyrosol in this context, we explore its effects on all the parameters studied. Our research helps shed light on the molecular mechanisms involved in the protective effects of hydroxytyrosol against Hg-induced vascular toxicity.

## 2. Materials and Methods

### 2.1. Reagents

The following reagents were used in this study. Physiological serum (NaCl 0.9%) (190/12626837/1221, Braun, Melsungen, Germany), Dulbecco’s PBS+ (DPBS+) (14040-091, Gibco, Boston, MA, USA), Dulbecco’s PBS-(DPBS-) (14190-094, Gibco), EBM-2 culture media (CC-3156, Lonza, Basel, Switzerland) supplemented with SingleQuots^®^ (CC-4176, Lonza) included fetal bovine serum, human serum albumin (HAS, albuminate 25%) (A9080, Sigma-Aldrich, Saint Louis, MI, USA), RPMI1640 (21875-034, Gibco) supplemented with 20 mmol/L HEPES (15630-056), HBSS without Ca^2+^ and Mg^2+^ (H6648, Sigma-Aldrich), fibronectin (F2006, Sigma-Aldrich), dextran (31392, Sigma-Aldrich), Ficoll-Paque^™^ Plus (17-1440-03, GE Healthcare, Chicago, IL, USA), PBS (20012-019, Gibco), collagenase (17018-029, Gibco) and 0.25% trypsin-EDTA 1× (25200-072, Gibco); Annexin-V (AD10) (Dojindo Laboratories, Kumamoto, Japan), dimethyl sulfoxide (DMSO) (D2650, Sigma-Aldrich), 3-hydroxytyrosol (91404, Sigma-Aldrich), mouse anti-human CD11b PE (555388, Lot: 1242226, BD Pharmingen™, Franklin Lakes, NJ, USA), mouse anti-human CD18 FITC (555923, Lot: 6238552, BD Pharmingen™), mouse anti-human CD11a APC-Cy7 (301236, Lot:B357935, BioLegend, San Diego, CA, USA), mouse anti-human PSGL-1(CD162) PerCp/Cy-5.5 (328818, Lot: B349883. BioLegend^®^), mouse anti-human CD62L PC7 (B30641, Lot: 200034, Beckman Coulter, San Diego, CA, USA), mouse anti-human ICAM-1 PE (555511, Lot: 0135026, BD Pharmingen™), mouse anti-human VCAM-1(CD106) FITC (551146, Lot: 2111025, BD Pharmingen™), mouse anti-human CD62P FITC (555523, Lot: 3191091, BD Pharmingen™), and mouse anti-human and CD62E PE (551145, Lot: 7180950, BD Pharmingen™).

### 2.2. Isolation of Erythrocytes and Leukocytes from Blood

The medical ethics committee of the Hospital Clínico Universitario de Valencia approved the use of human materials for the study (2021/038), and all participating patients provided their written informed consent. The study was conducted in accordance with the principles of the Declaration of Helsinki.

For the extraction of RBCs, whole blood was collected in heparinized tubes and centrifuged at 2000 rpm for 10 min at 4 °C. After removing the buffy coat, the RBC fraction was washed twice with isotonic saline solution (0.9% NaCl) and resuspended in Krebs solution at pH 7.4 containing (in mM) NaCl 125, KCl 4, MgSO_4_ 1, Hepes 32, CaCl_2_ 1, and glucose 5 [5].

To extract PMNs, whole blood, anticoagulated with sodium citrate, was collected from healthy volunteers, none of whom had been exposed to any anti-inflammatory drug in the previous 15 days. Whole blood samples were incubated with dextran (3% in physiological serum) for 45 min. Subsequently, PMNs in the supernatant were separated using density gradient centrifugation (2500 rpm for 25 min) with Ficoll-Paque™ Plus. After RBC lysis, PMNs were washed twice with HBSS without Ca^2+^ and Mg^2+^ (1200 rpm for 5 min) and resuspended in complete RPMI medium [22].

### 2.3. Human Umbilical Vein Endothelial Cell Culture

To isolate human umbilical vein endothelial cells (HUVECs), umbilical cord veins were rinsed with a warm PBS solution and then filled with collagenase solution (1 mg/mL) for 17 min at 37 °C. The cords were then gently massaged to detach the endothelial cells from the vessel walls. The resulting digest was collected and centrifuged at 1200 rpm for 5 min. The pellet was then resuspended in endothelial basal medium (EBM-2) in T-25 cm^2^ culture flasks, where the cells were grown to confluence. After reaching confluence, the primary cultures were detached with trypsin and transferred to 6-well culture plates. For the adhesion studies, HUVECs were grown on 25 mm plastic coverslips coated with fibronectin (5 μg/mL) until they reached confluence (approximately 48 h).

### 2.4. Experimental Protocol

Subsequently, samples were incubated with different concentrations of HgCl_2_ (1–20 μM) or vehicle (DMSO) for either 4 h (RBCs and PMNs) or 24 h (HUVECs) or with different positive stimuli, namely PMNs with phorbol myristate acetate (PMA, 2.5 ng/mL) and HUVECs with TNF-α (25 ng/mL). In some cases, cells were pre-treated, 15 min earlier, with different concentrations of hydroxytyrosol (20–40 μM).

### 2.5. Interactions of Leukocytes and Erythrocytes with Endothelial Cells Under Flow Conditions

The parallel plate flow chamber system was used as previously described to evaluate the adhesion of PMNs or RBCs to HUVECs [23]. In brief, coverslips with confluent monolayers of HUVECs were placed in a chamber at 37 °C, and a portion (5 mm × 25 mm) was exposed to the flow. The chamber was mounted on an inverted microscope (Nikon Eclipse TE 2000-S, 40× magnification; Amstelveen, The Netherlands) connected to a video camera (Sony Exware HAD; Cologne, Germany).

RBCs were resuspended in a Krebs solution with a final hematocrit of 0.2%, while PMNs were prepared at a concentration of 1 × 10^6^ cells/mL in a flow buffer (Dulbecco’s PBS, with and without Ca^2+^ and Mg^2+^ containing 20 mmol/L HEPES and 0.1% HSA). Subsequently, RBCs or PMNs were allowed to flow over a HUVEC monolayer at a controlled rate of 0.36 mL/min (with an estimated shear stress of 0.7 dyne/cm^2^) to perform RBC–HUVEC or PMN–HUVEC adhesion assays, respectively. Single-field images were recorded for 5 min.

In the case of RBC–HUVEC adhesion assays, the number of RBC cells that maintained stable contact with the HUVEC monolayer for 30 s was measured [10].

For PMN–HUVEC assays, the rolling flow of PMNs was calculated by counting the number of cells rolling across 100 μm^2^ of the monolayer in one minute. The speed of 20 consecutive PMNs in the field of view was analyzed by measuring the time required to travel 100 μm. PMN adhesion was quantified after 5 min of perfusion by analyzing 5–10 high-power fields (40× magnification). PMNs were considered to be adherent after 30 s of stable contact with the monolayer.

### 2.6. Analysis of the Expression of Adhesion Molecules and Phosphatidylserine

After the treatments, PMNs or HUVECs were centrifuged at 1200 rpm for 5 min and resuspended in PBS. Afterward, PMNs were incubated in the dark for 20 min at 4 °C with antibodies against CD11b, CD11a, CD18, PSGL-1 (CD162), and L-selectin (CD62L), while HUVECs were incubated with those against ICAM-1, VCAM-1, P-selectin (CD62P), and E-selectin (CD62E), conjugated with either PE or FITC.

Following the treatment, in order to analyze PS expression, PMNs were centrifuged twice for 3 min at 1200 rpm, resuspended in 100 μL of 1× binding buffer, and incubated in the dark for 15 min at room temperature with 2.5 μL of Annexin-V.

The analysis was performed using a Cytoflex B5-R3-V0 (Beckman Coulter). The median fluorescence intensity was observed, and fluorescence values were expressed as a percentage of the mean of the median fluorescence intensity vs. the vehicle (DMSO). Nonspecific fluorescence was detected using isotype-matched nonbinding antibodies, the value of which was subtracted from that obtained in each experimental group. Experiments were performed in duplicates, and 10,000 cells were analyzed in each sample.

### 2.7. Statistics

All the procedures performed complied with Spanish law. Data are presented as the mean ± S.E.M. (*n* ≥ 4). Statistical analyses were performed (GraphPad Prism 10.0) by a person blinded to the experimental conditions. One-way analysis of variance (ANOVA) with a Newman–Keuls post-hoc correction for multiple comparisons was performed. The following values of * *p* < 0.05, ** *p* < 0.01 or *** *p* < 0.001 indicate statistical significance vs. the corresponding value in the vehicle-treated group, while # *p* < 0.05 or ## *p* < 0.01 indicate statistical significance vs. the corresponding value in the HgCl_2_-treated group.

## 3. Results

### 3.1. Analysis of Erythrocyte and Leukocyte Adhesion to Endothelial Cells

**Figure 1:**
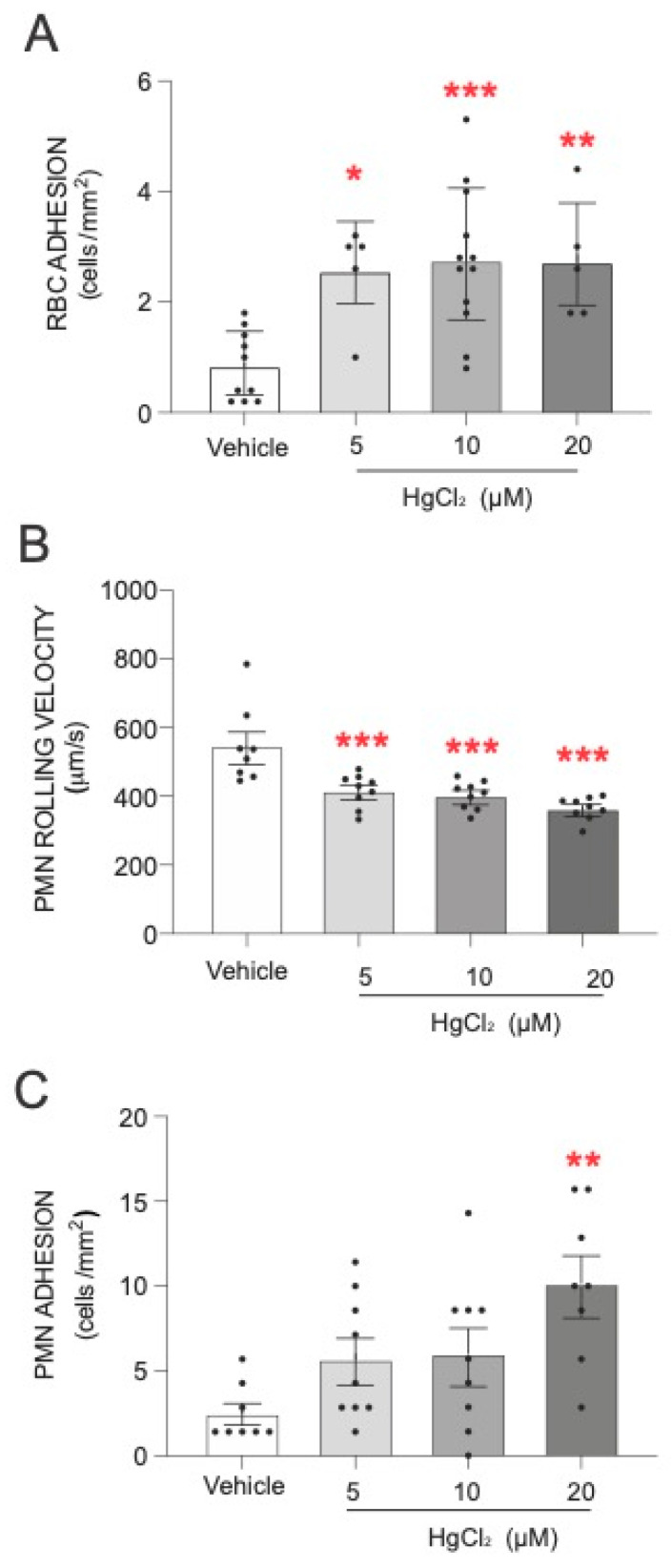
Effects of HgCl_2_ on RBCs or PMN–endothelial-cell interactions. RBCs (Red Blood Cells), PMNs (Polymorphonuclear cells), and/or HUVECs (Human Umbilical Vein Endothelial cells) were incubated (RBCs/PMNs for 4 h and HUVECs for 24 h) with HgCl_2_ (5–20 µM) or a vehicle (DMSO). After assembling the flow chamber, we evaluated the interactions of RBCs (**A**) and PMNs [rolling velocity (**B**) or adhesion (**C**)] with HUVECs. Data are presented as the mean ± S.E.M. (*n* ≥ 4). * *p* < 0.05, ** *p* < 0.01 or *** *p* < 0.001 indicate statistical significance vs. the corresponding value in the vehicle-treated group (ANOVA, followed by Newman–Keuls test).

The first step of our study was to evaluate the effect of HgCl_2_ on the adhesion of RBCs and PMNs to HUVECs. As shown in Figure 1, treatment of RBCs with HgCl_2_ led to a significant increase in RBC adhesion to endothelial cells (Figure 1A). An alteration was produced under all the concentrations of HgCl_2_ tested. In addition, we evaluated the effect of HgCl_2_ on PMN–endothelium interactions. Our results demonstrate that treatment with HgCl_2_ caused a dose-dependent significant reduction in the rolling velocity of PMNs (Figure 1B) and an increase in the adhesion (Figure 1C) of PMNs to endothelial cells.

### 3.2. Analysis of Leukocyte and Endothelial Adhesion Molecules and Phosphatidylserine Expression

To assess the molecular processes underlying these interactions, we analyzed the expression of various adhesion molecules in isolated HUVECs (Table 1) using flow cytometry. The highest dose of HgCl_2_ (20 µM) had no effect on the expression of any of the endothelial cells (Table 1).

**Figure 2:**
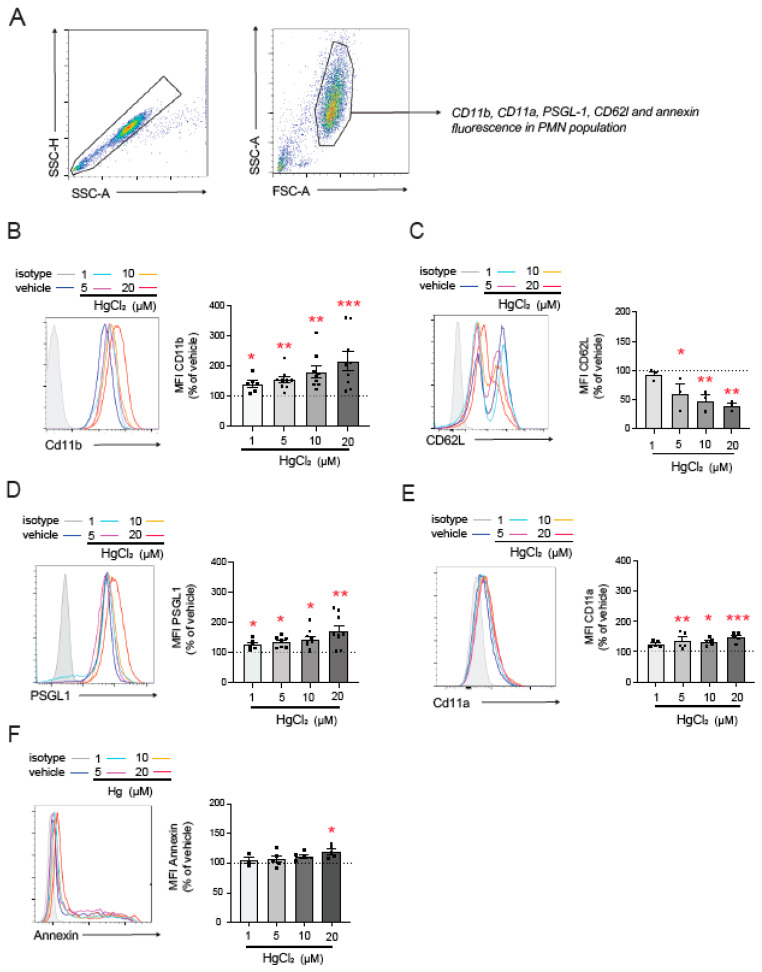
Effect of HgCl_2_ on the expression of adhesion molecules in PMNs. PMNs (Polymorphonuclear cells) were treated (4 h) with HgCl_2_ (1 µM, 5 µM, 10 µM, and 20 µM) or a vehicle and the expression of the adhesion molecules CD11b (**B**), CD62L (**C**), PSGL-1 (**D**), and CD11a (**E**) and of the extracellular side of the membrane phospholipid PS (**F**) was measured by flow cytometry following the gating strategy shown in (**A**). Fluorescence values are expressed as a percentage of median fluorescence intensities of the control cells (vehicle, dotted line). Data are presented as the mean ± S.E.M. (*n* ≥ 4). * *p* < 0.05, ** *p* < 0.01 or *** *p* < 0.001 indicate statistical significance vs. the corresponding value in the vehicle-treated group (ANOVA followed by Newman–Keuls test). PSGL-1: P-selectin glycoprotein ligand-1.

To further investigate the mechanisms involved in HgCl_2_-induced PMN–endothelium interactions, we, once again, used flow cytometry to explore the effects of HgCl_2_ on the main adhesion molecules expressed by PMNs (Figure 2A). HgCl_2_ exposure significantly increased CD11b expression (Figure 2B) and decreased that of L-selectin (CD62L, Figure 2C)—both markers of PMN activation—in a dose-dependent manner. Moreover, the HgCl_2_ treatment resulted in the overexpression of the adhesion molecules PSGL-1 (Figure 2D) and CD11a (Figure 2E).

Given the relevance of PS in the interactions between RBCs and the endothelium [5], we evaluated PS exposure on the outer membrane surface of PMNs; by doing this, we aimed to determine which molecules mediate PMN–HUVEC interactions. We observed an increase in PS exposure compared to physiological conditions after the treatment with high concentrations of HgCl_2_ (20 µM), while no changes were observed at lower concentrations.

### 3.3. Protective Effect of Hydroxytyrosol on Mercury-Induced Adhesion of Erythrocytes and Leukocytes to Endothelial Cells

**Figure 3:**
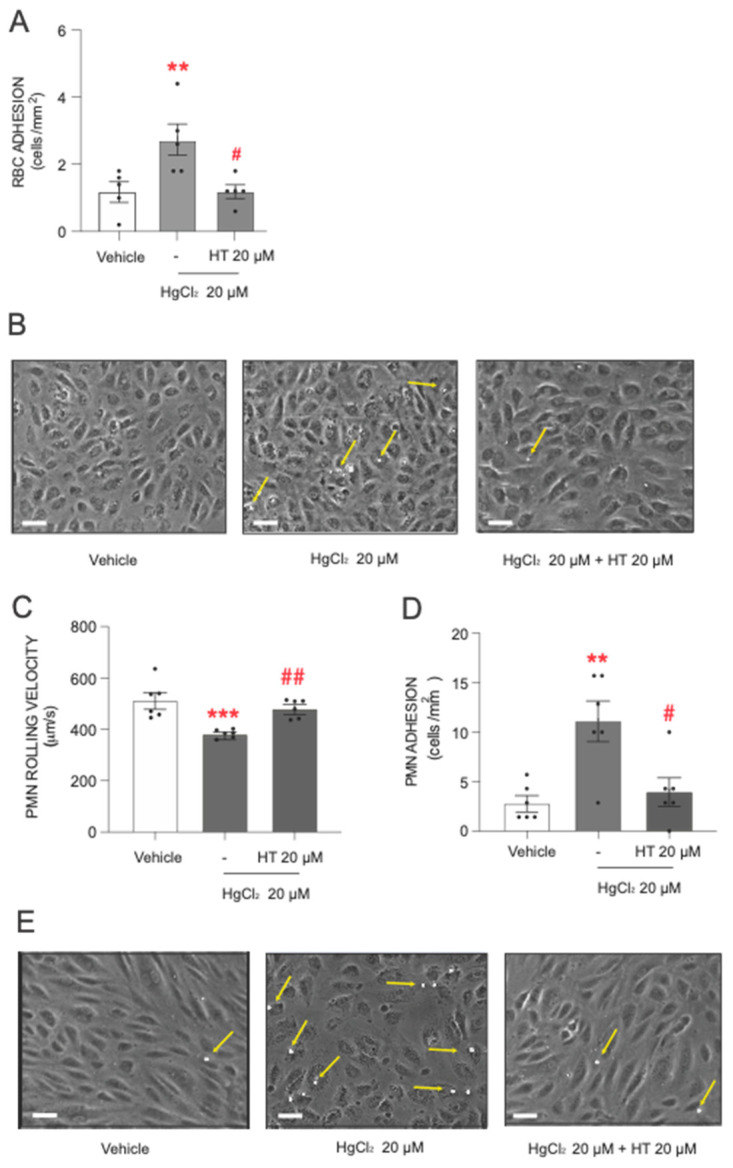
Protective effects of hydroxytyrosol on HgCl_2_-induced RBC or PMN adhesion to endothelial cells. RBCs (Red Blood Cells), PMNs (Polymorphonuclear cells), and/or HUVECs (Human Umbilical Vein Endothelial cells) were incubated (RBCs/PMNs for 4 h and HUVECs for 24 h) with HgCl_2_ 20 µM) or a vehicle (DMSO). In some cases, cells were pretreated with hydroxytyrosol (HT) 20 µM 15 min prior to the HgCl_2_ treatment. After assembling the flow chamber, we evaluated the interactions of RBCs (**A**) or PMNs [rolling velocity (**C**) or adhesion (**D**)] with HUVECs. (**B**,**E**) are representative images of RBC– and PMN–endothelial-cell interactions, respectively. Scale bar: 100 µm. Yellow arrows indicate RBC or PMN adhere to HUVECs. Data are presented as the mean ± S.E.M. (*n* ≥ 4). ** *p* < 0.01 or *** *p* < 0.001 indicate statistical significance vs. the corresponding value in the vehicle-treated group, while # *p* < 0.05 or ## *p* < 0.01 indicate statistical significance vs. the corresponding value in the HgCl_2_-treated group (ANOVA followed by Newman–Keuls test).

After describing the toxic actions of HgCl_2_, the next step was to assess the protective effect of hydroxytyrosol. The data showed that pretreatment with hydroxytyrosol significantly reduced the level of adhesion of RBCs and PMNs to endothelial cells induced by the heavy metal (Figure 3). The influence of hydroxytyrosol was particularly pronounced in RBCs (Figure 3A,B), where a protective effect was observed at the highest HgCl_2_ concentration (20 µM) tested.

Regarding PMN adhesion, hydroxytyrosol exerted a statistically significant protective effect when PMNs were treated with 20 µM of HgCl_2_. Under these conditions, we observed a significant increase in PMN rolling velocity (Figure 3C) and a drastic reduction in the number of PMN cells adhering to the endothelial wall (Figure 3D,E), with both parameters reverting almost to near physiological levels.

### 3.4. Protective Effect of Hydroxytyrosol Against the Mercury-Induced Expression of Adhesion Molecules and Phosphatidylserine on Leukocytes

**Figure 4:**
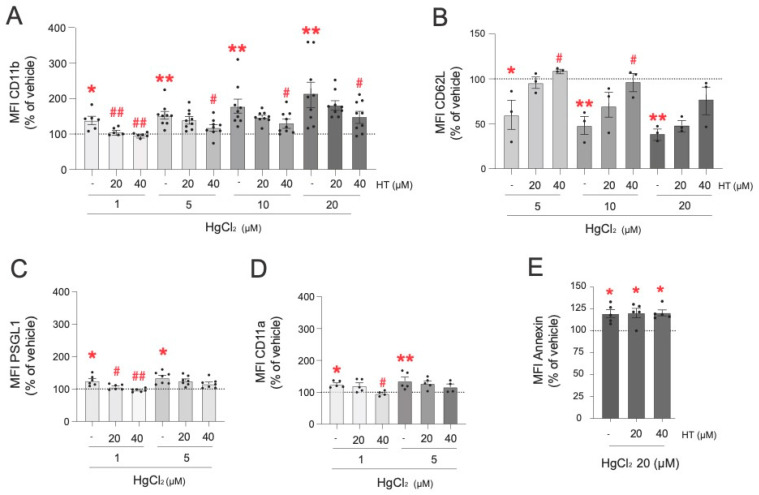
Protective effects of hydroxytirosol on the expression of adhesion molecules in PMNs induced by HgCl_2_. PMNs (Polymorphonuclear cells) were treated (4 h) with HgCl_2_ (1 µM, 5 µM, 10 µM, and 20 µM) or with a vehicle. In some cases, cells were pretreated with hydroxytyrosol (HT) 20 µM or 40 µM 15 min before the HgCl_2_ treatment. Subsequently, the expression of the adhesion molecules CD11b (**A**), CD62L (**B**), PSGL-1 (**C**), and CD11a (**D**) and of the extracellular side of the membrane phospholipid PS (**E**) were measured by flow cytometry. Fluorescence values are expressed as a percentage of the median fluorescence intensities of the control cells (vehicle, dotted line). Data are presented as the mean ± S.E.M. (*n* ≥ 4). * *p* < 0.05, ** *p* < 0.01 indicate statistical significance vs. the corresponding value in the vehicle-treated group, while # *p* < 0.05 or ## *p* < 0.01 indicate statistical significance vs. the corresponding value in the HgCl_2_-treated group (ANOVA followed by Newman–Keuls test).

Subsequently, we explored the protective effect of hydroxytyrosol on HgCl_2_-induced alterations to the expression of PMN adhesion molecules (Figure 4). The highest dose of hydroxytyrosol (40 µM) was highly effective in preventing CD11b expression induced by all the concentrations of HgCl_2_ tested (1 µM, 5 µM, 10 µM, and 20 µM) (Figure 4A). Importantly, when PMNs were treated with lower concentrations of HgCl_2_ (1 µM), the presence of hydroxytyrosol (20 µM and 40 µM) restored the concentrations found under physiological conditions. In the case of CD62L expression, only the highest dose of hydroxytyrosol (40 µM) significantly restored normal expression levels after treatment with 5 µM and 10 µM of HgCl_2,_ but not after treatment with 20 µM of HgCl_2_ (Figure 4B). Regarding PSGL-1 (Figure 4C), pretreatment with hydroxytyrosol at concentrations of 20 µM or 40 µM restored normal levels of this adhesion molecule in PMNs when they were treated with 1 µM of HgCl_2_, but no protective effects were detected at the HgCl_2_ concentration of 5 µM. A similar scenario was observed with respect to CD11a expression; hydroxytyrosol exerted a protective effect only at a concentration of 40 µM and only when low concentrations of HgCl_2_ (1 µM) were employed (Figure 4D). Finally, none of the hydroxytyrosol concentrations evaluated (20 µM and 40 µM) restored the PS levels modified by either of the two Hg concentrations tested (10 µM and 20 µM) (Figure 4E).

### 3.5. Protective Influence of Hydroxytyrosol on the Effects of Other Positive Stimuli

We decided to evaluate the protective effect of hydroxytyrosol on the previously described parameters altered by other positive stimuli. To do this, RBCs and PMNs were treated with PMA, while HUVECs were treated with TNFα (both referred to, from now on, as “positive stimuli”). The positive stimuli increased RBC (Supplementary Figure S1A) and PMN adhesion (Supplementary Figure S1B) to the endothelium. In both cases, hydroxytyrosol (20 µM) reduced the adhesion of RBCs (Supplementary Figure S1A) and PMNs (Supplementary Figure S1B) to the endothelial wall induced by the positive stimuli.

The expression of adhesion molecules in PMNs and HUVECs was also evaluated. In PMNs, the PMA treatment led to a significant increase in CD11b (Supplementary Figure S1C) and PSGL1 (Supplementary Figure S1D) and to a decrease in L-selectin (Supplementary Figure S1E), effects that were significantly reversed when the cells were pretreated with hydroxytyrosol (20 µM). TNFα stimulation increased the expression of the endothelial adhesion molecules ICAM-1 (Supplementary Figure S1F), CD62P (Supplementary Figure S1G), VCAM-1 (Supplementary Figure S1H), and CD62E (Supplementary Figure S1I); however, hydroxytyrosol did not modify said expression levels, suggesting that it had no protective effects on these cells.

## 4. Discussion

Adhesion of RBCs and/or PMNs to the vascular endothelium is a key stage in the development and progression of CVDs, as it is implicated in inflammation, atherosclerotic plaque formation, blood viscosity, and thrombus formation [24,25]. Therefore, identifying molecules that impede this process is an essential part of developing effective preventative and therapeutic strategies. Several studies have highlighted the effectiveness of natural products, including phenolic compounds from olive oil—one of which is hydroxytyrosol—in preventing various types of cancer and neurodegenerative diseases like Parkinson’s and Alzheimer’s [26,27,28]. Recently, numerous experimental and clinical studies have demonstrated the beneficial health effects of hydroxytyrosol. Notably, its protective cardiovascular and anti-inflammatory properties against toxic agents, including Hg, have been attributed to its ability to modulate cellular responses such as RBC–endothelium interactions [29,30]. However, the underlying molecular mechanisms through which hydroxytyrosol exerts these protective cardiovascular effects are yet to be identified.

**Figure 5:**
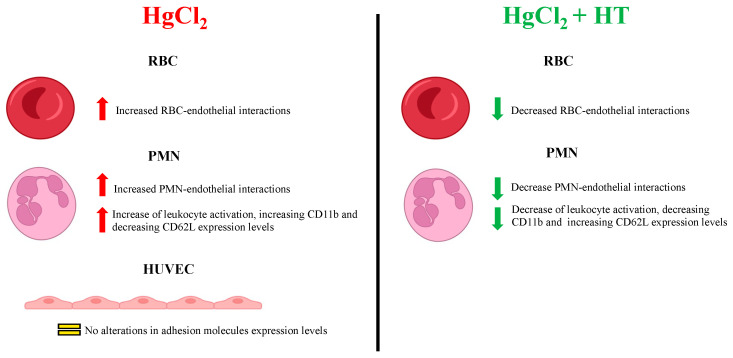
Summary of the effects of HgCl_2_ on the different cell types evaluated alongside the protective effects of hydroxytirosol.

Our data indicate that Hg induces a significant increase in the adhesion of RBCs and PMNs to the endothelium, findings which may explain why intoxication with this metal is associated with the development of CVDs. We did not observe changes in the expression of the endothelial adhesion molecules (ICAM-1, VCAM-1, CD62E, and CD62P) after treatment with HgCl_2_ (Figure 5). Therefore, in the context of RBC–endothelium interactions, PS exposure might represent a key molecular mechanism underlying the increased adhesiveness of RBCs to the endothelium, as proposed by Notariale et al. [5]. In addition, an increase in the expression of the PS receptor (PSR) in endothelial cells—crucial for the adhesion of various cell types to the endothelium—cannot be ruled out. Indeed, Setty et al. showed that the activation of endothelial cells through various stimuli, such as TNFα, leads these cells to express high levels of this receptor on their surface [31].

Regarding PMN–endothelium interactions induced by Hg, we observed that this metal activates PMNs by inducing the expression of CD11b, PSGL-1, CD11a, and PS and by downregulating L-selectin in a dose-dependent manner (Figure 5). Our data align with those from recent studies showing that exposure of human microvascular endothelial cells-1 (HMEC-1) to methylmercury (MeHg) causes a significant increase in monocyte binding through the expression of different adhesion molecules and chemokines [18].

Next, we evaluated the protective effects of hydroxytyrosol on HgCl_2_-induced RBC and PMN adhesion to human endothelial cells. We observed that hydroxytyrosol exerts a significant protective effect by decreasing RBC adhesion to HUVECs. These results align with those from previous studies demonstrating how hydroxytyrosol reduces PS levels on RBCs’ membrane following HgCl_2_ treatments [5]. Therefore, even though we observed no changes in conventional adhesion molecules, hydroxytyrosol may well reduce RBC cellular adhesion by impeding PS distribution rather than directly modifying the expression of adhesion molecules.

In relation to PMN–endothelium interactions, we demonstrate that hydroxytyrosol protects against HgCl_2_-induced neutrophil activation and adhesion to the endothelium. Hydroxytyrosol acts on PMN and RBC interactions with the endothelium in different ways. Our data indicate that the protective effect of hydroxytyrosol on PMN–endothelium interactions is a result of the blockade of the expression of different adhesion molecules. This effect would appear to be independent of PS expression, which is responsible, particularly, for the binding of RBCs to the vascular endothelium, as mentioned above. In fact, we provide evidence of the protective effect of hydroxytyrosol on RBC–endothelium and PMN–endothelium interactions and on neutrophil activation beyond the context of Hg treatment; to be precise, we show that hydroxytyrosol protects against the proinflammatory effect of PMA, a well-known inducer of neutrophil activation, but it cannot block the effect of TNFα stimulation on HUVECs.

Our results offer new insights into the protective role of hydroxytyrosol in reducing RBC and PMN adhesion to endothelial cells, particularly in the context of Hg toxicity. This is of particular clinical interest, since RBC–endothelium and PMN–endothelium interactions play an important role in the development of complications such as thrombosis in polycythemia vera and splenic sequestration in hereditary spherocytosis [32]. RBC adhesion to the vascular endothelium may be also involved in the pathogenesis of vaso-occlusive crises associated with the sickle cell disease. Interestingly, recent data suggest that abnormal PS-mediated RBC adhesion is involved in the pathophysiology of non-erythroid disorders, which share common clinical manifestations (including thrombotic events) such as central retinal vein occlusion and Gaucher disease [33,34].

It is relevant that different toxic molecules are known to induce alterations to RBC membranes, particularly those related to the redistribution of PS. Recent studies have shown that PS exposure is also promoted by the presence of uremic toxins. Hamza et al. described this phenomenon as one of the main effects of such toxins, and associated it with anemia in patients suffering from chronic kidney disease [35]. Furthermore, the disruption of normal membrane asymmetry is associated with the action of various bacterial toxins. Specifically, Jewell et al. demonstrated how the toxin produced by *Clostridium perfringens* interacts with erythrocytes, leading to the remodeling of the membrane [36].

This study has several notable strengths. Among them, the detailed approach adopted to evaluate the toxic effects of HgCl_2_ at both cellular and molecular levels stands out, as it integrates functional analyses (adhesion and rolling of RBCs and PMNs) with molecular analyses (expression of adhesion molecules and PS). Additionally, the inclusion of hydroxytyrosol as a protective agent provides a potential therapeutic intervention, which is particularly relevant to mitigate Hg toxicity that can be exacerbated in the presence of other stimuli. However, there are some limitations. The study focuses primarily on in vitro models (HUVECs, isolated RBCs, and PMNs), which may not fully replicate the complex interactions occurring in a living organism. Moreover, the efficacy of HT is demonstrated mainly for specific molecules and under certain conditions, suggesting the need for further studies to validate these effects in a broader and in vivo context.

In conclusion, the present study provides new insights into how hydroxytyrosol mitigates the harmful effects of heavy metals (Figure 5), thus endorsing it as a component of nutritional and nutraceutical strategies to counteract the clinical outcomes of chronic Hg exposure in humans, particularly in the context of the prevention of CVDs. Future research should focus on exploring the molecular mechanisms through which hydroxytyrosol exerts its protective effects in order to move forward in the development of effective interventions against CVDs and other inflammation-related diseases.

## Figures and Tables

**Table 1:** Effect of HgCl_2_ on the expression of endothelial adhesion molecules. HUVECs (Human Umbilical Vein Endothelial cells were treated) (24 h) with HgCl_2_ (20 µM) or a vehicle and the expression of the adhesion molecules ICAM 1, VCAM-1, E-selectin, and P-selectin was measured by flow cytometry in HUVECs.

	HgCl_2_ 20 µM (% of Vehicle ± S.E.M.)
ICAM-1	101.7 ± 5.7
VCAM-1	104.0 ± 7.7
P-selectin	110.0 ± 8.5
E-selectin	112.0 ± 11.0

## Data Availability

Inquiries regarding the original contributions presented in the study can be directed to the corresponding author.

## Supplementary Materials

The following supporting information can be downloaded at: https://www.mdpi.com/article/10.3390/antiox13121576/s1, Figure S1: Protective effects of hydroxytirosol on blood cell interactions and the expression of adhesion molecules induced by positive stimuli.
