# Supplementary material for: Increased Adhesiveness of Blood Cells Induced by Mercury Chloride: Protective Effect of Hydroxytyrosol
Source: Antioxidants (Basel). 2024 Dec 20;13(12):1576. doi: 10.3390/antiox13121576 (PMC11673208; doi:10.3390/antiox13121576)
Supplement: Supplementary file 1 [file antioxidants-13-01576-s001.zip › antioxidants-3337691-supplementary.pdf]

Supplementary material

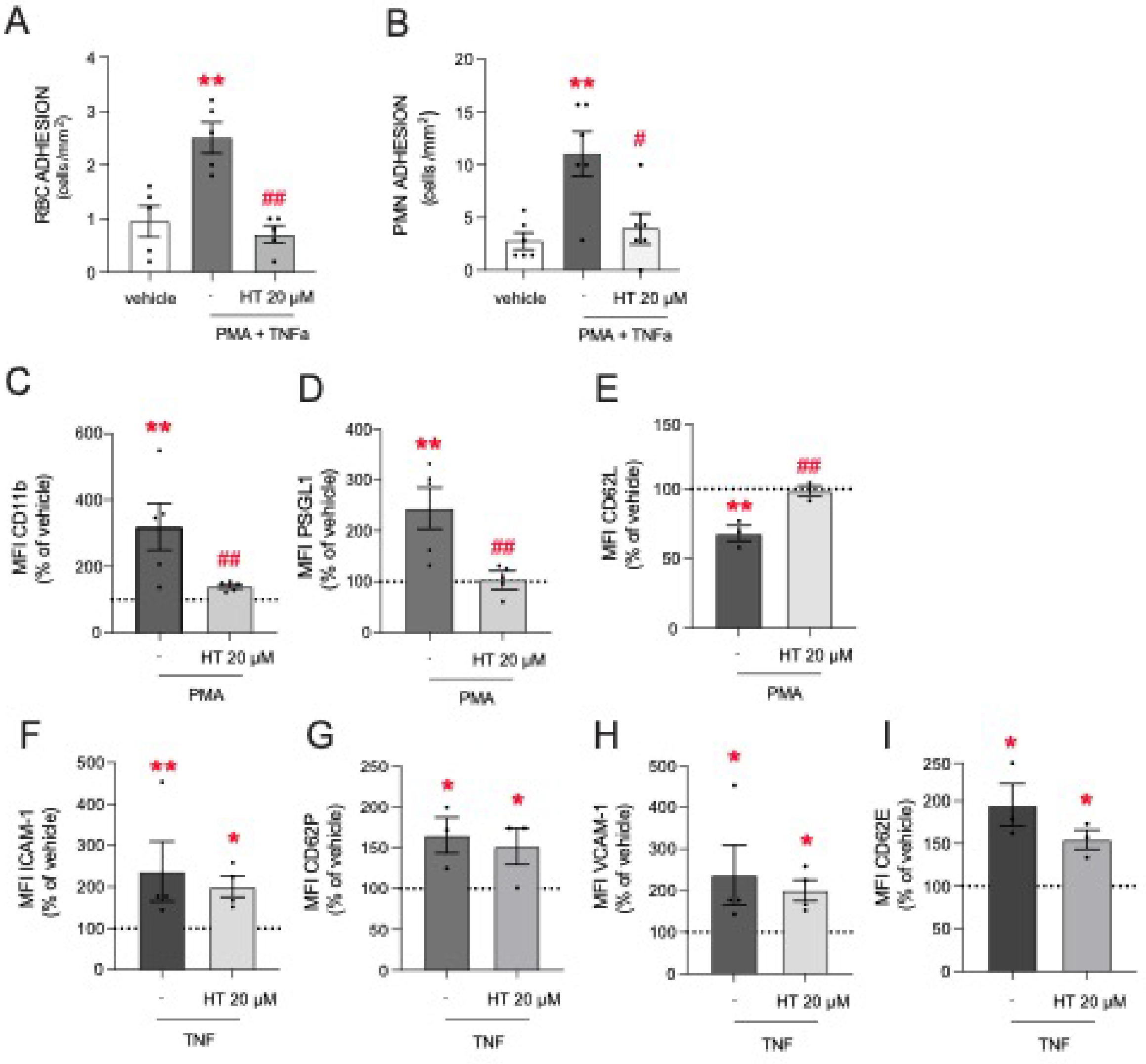

Supplementary figure S1. Protective effects of Hydroxytyrosol on blood cell interactions and the expression of adhesion molecules induced by positive stimuli. RBC or PMN were incubated (4h) with PMA (2.5 ng/mL) and HUVEC (24h) with TNF-α (25 ng/mL). In some cases, cells were pretreated with hydroxytyrosol (HT) 20 μM 15 min prior to the stimuli. After assembling the flow chamber, we evaluated the adhesion of RBC (A) or PMN (B) to HUVEC. For flow cytometry analysis, PMN or HUVEC cells received the above mentioned treatments and the expression of the adhesion molecules CD11b (C), PSGL-1 (D) and CD62L (E) were measured in the PMN population, while that of ICAM-1 (F), CD62P (G), VCAM-1 (H) and CD62E (I) were analyzed in HUVEC. Fluorescence values are expressed as a percentage of the median fluorescence intensities of control cells (vehicle, dotted line). Data are presented as mean ± S.E.M. (n ≥ 4). * p<0.05, ** p<0.01 or *** p<0.001 indicate statistical significance vs. corresponding value in vehicle-treated group, while #p<0.05 or ## p<0.01 indicate statistical significance vs. corresponding value in the $HgCl_2$-treated group. (ANOVA followed by Newman-Keuls test).
